# Study of the Etiological Causes of Toe Web Space Lesions in Cairo, Egypt

**DOI:** 10.1155/2015/701489

**Published:** 2015-09-21

**Authors:** Hussein Mohamed Hassab-El-Naby, Yasser Fathy Mohamed, Hamed Mohamed Abdo, Mohamed Ismail Kamel, Wael Refaat Hablas, Osama Khalil Mohamed

**Affiliations:** ^1^Al-Hussein University Hospital, Gawhar Al Qaed Street, El Darrasa, Cairo 11633, Egypt; ^2^Dermatology, Venereology and Andrology, Faculty of Medicine, Al-Azhar University, Cairo 11823, Egypt; ^3^Clinical and Chemical Pathology, Faculty of Medicine, Al-Azhar University, Cairo 11823, Egypt

## Abstract

*Background*. The etiology of foot intertrigo is varied. Several pathogens and skin conditions might play a role in toe web space lesions. *Objective*. To identify the possible etiological causes of toe web space lesions. *Methods*. 100 Egyptian patients were enrolled in this study (72 females and 28 males). Their ages ranged from 18 to 79 years. For every patient, detailed history taking, general and skin examinations, and investigations including Wood's light examination, skin scraping for potassium hydroxide test, skin swabs for bacterial isolation, and skin biopsy all were done. *Results*. Among the 100 patients, positive Wood's light fluorescence was observed in 24 and positive bacterial growth was observed in 85. With skin biopsy, 52 patients showed features characteristic for eczema, 25 showed features characteristic for fungus, 19 showed features characteristic for callosity, and 3 showed features characteristic for wart while in only 1 patient the features were characteristic for lichen planus. *Conclusion*. Toe web space lesions are caused by different etiological factors. The most common was interdigital eczema (52%) followed by fungal infection (25%). We suggest that patients who do not respond to antifungals should be reexamined for another primary or secondary dermatologic condition that may resemble interdigital fungal infection.

## 1. Introduction

Toe web intertrigo may present as a relatively asymptomatic, mild scaling but it also can be seen as a painful, exudative, macerated, erosive, inflammatory process which is sometimes malodorous [1].

Foot intertrigo is mostly caused by dermatophytes and yeasts and less frequently by Gram-negative and Gram-positive bacteria. Gram-negative infection is relatively common and may represent a secondary infection of tinea pedis. With time, a “complex” may develop in the setting of moisture and maceration that contains multiple fungal and bacterial organisms [2, 3]. Frequently, these interdigital lesions are often diagnosed as tinea pedis or eczematous dermatitis. However, in some patients, the macerated eruption is unresponsive to treatment with antifungal agents or anti-inflammatory agents such as topical steroids [4].

Other less common conditions may also affect the web space such as erythrasma [5]. Because the texture of soft corn is macerated, it may be misdiagnosed clinically as a mycotic infection of the interdigital space [6]. Interdigital psoriasis (“white psoriasis” or “psoriasis alba”) is a distinct but atypical form of psoriasis that is often missed as it is commonly mistaken for interdigital fungal infection [7]. A report of Bowen's disease [8] and a case of* Verrucous* carcinoma in the third and fourth toe web space which is presented by intractable intertrigo has been reported [9]. Also, malignant melanoma in the interdigital space has been diagnosed [10].

It is apparent that several pathogens and factors might play a role in toe web space lesions. Therefore, clinical and microbiologic studies are suggested to assist in the selection of appropriate treatment and the prevention of important complications [3]. In this study, we aimed to determine the different etiological causes of pedal web space lesions.

## 2. Patients and Methods

This study included 100 Egyptian patients living in Cairo who presented at Dermatology Departments of Al-Azhar University Hospitals with toe web space lesions. The study was approved by the Al-Azhar University Medical Ethics Committee, and written prior informed consent was obtained from every participant. Patients who have received systemic and/or topical antifungal and/or antibiotic treatments in the past 6 weeks were excluded.

Every patient was subjected to the following: (1) full history taking; (2) general and local examinations; (3) Wood's light examination for possible fluorescence; (4) skin scraping for direct potassium hydroxide (KOH) test: scraping of lesions was done with a blunt scalpel then a drop of 20% KOH/40% dimethyl sulfoxide (DMSO) mixture was added to the specimen (DMSO increases sensitivity of the preparation and softens keratin more quickly than KOH alone in the absence of heat [11]); the slide was then examined for fungus using low power field ×10 then high power field ×40; (5) skin swabs for bacterial isolation: cotton swabs were taken from the lesions and incubated for 24 hours at 37°C in blood and MacConkey (BIOTEC Lab. Ltd., UK) and nutrient (Oxoid Ltd., England) agars; the bacterial cultures were considered negative if there was no growth after 48 hours of incubation; the suspected colony was picked up for morphological and biochemical reactions for their identifications; (6) a 3 mm punch biopsy was taken from the lesion. Specimens were fixed in 10% formalin then sectioned and stained with haematoxylin and eosin stain (as a routine) and PAS stain (to highlight fungal elements).

All involved toe web spaces were examined with Wood's light while KOH test, skin swabs for bacterial isolation, and skin biopsy were done from the most affected web space showing intensive erythema and desquamation (almost the 4th web space). Wood's light examination aimed to show the characteristic coral red fluorescence with erythrasma [12].

The goal of mycological workup in this study was to demonstrate just the presence of fungi (regardless of its nature) as an etiologic cause of web space lesions; so we thought that KOH test (supported by PAS-stained tissue sections) will suffice this aim. The recognition of fungal organisms as dermatophyte, mould, or yeast by KOH is presumptive although it is highly probable [13]. We relied upon the WHO guidelines on standard operating procedures for microbiology [14]. These guidelines document that dermatophyte has regular, small hyphae (2-3 *μ*m) with some branching, sometimes with rectangular arthrospores.* Candida* has hyphae/pseudohyphae (with distinct points of constriction) with budding yeast forms.* Aspergillus* species had hyphae that are usually small (3–6 *μ*m) and regular in size, dichotomously branching at 45-degree angles with distinct cross septa.

The histopathological examination aimed to correlate with and confirm the clinical diagnosis. A diagnosis of lichen planus is established when the biopsy showed the following criteria: dense, band-like infiltrate of lymphocytes that is strictly confined to the subepidermal area. The lymphocytes attack and destroy the basic part of the epidermis, giving rise to characteristic sawtooth like rete ridges. The cell infiltrate in the dermis consists of lymphocytes with a mixture of some mast cells and macrophages; there is also a variable amount of melanin pigment which has leaked from the injured epidermis. There are no plasma cells or eosinophils [15].

In biopsies stained with PAS, fungal elements are highlighted, with hyphae and spores stained red. Dermatophytes produce septate hyphae and arthrospores [15].

Biopsy from patients with eczema demonstrates that epidermis shows moderate to marked acanthosis and hyper/parakeratosis. There may be areas of inter- and intracellular edema and rarely scattered small vesicles. The inflammatory cell infiltrates mainly consist of lymphocytes. Edema in the dermis is not prominent. Sometimes the pattern is psoriasis-like and shows long, slender rete ridges and papillae, which are covered by thin epidermis and contain thin-walled dilated venules filled with erythrocytes [15].

Acanthosis, papillomatosis, and hyperkeratosis are observed in biopsies from patients with warts, with confluence of the epidermal ridges in the centre of the lesion and koilocytes [16].

A thickened compact stratum corneum with slight cup-shaped depression of the underlying epidermis is seen in patients with callosity. The granular layer, in contrast to corn, may be thickened. There may be some parakeratosis overlying the dermal papillae, but much less than in a corn [17].

## 3. Results

### 3.1. Demographic and Clinical Findings

Of 100 patients with toe web space lesions, 72 were females and 28 were males. Their ages ranged from 18 to 79 years (mean 43.94 years; SD ±14.94). Regarding the presenting symptoms, 64 patients complained of pruritus while only 2 complained of pain and 34 were asymptomatic. The 4th web space was the commonest space affected in both right and left feet, followed by 3rd space, 2nd space, and 1st space (79%, 62%, 33%, and 7% versus 76%, 63%, 30%, and 8%, resp.) (Table 1). Of the 100 patients, 8 patients had lesion in 1 web space, 21 had lesion in 2 web spaces, 26 had lesion in 3 web spaces, 17 had lesion in 4 web spaces, 6 had lesion in 5 web spaces, 18 had lesion in 6 web spaces, 1 had lesion in 7 toe web spaces, and 3 had lesion in 8 web spaces.

### 3.2. Wood's Light Examination

Among the 100 patients, positive Wood's light fluorescence was observed in 24; of them, 20 were females and 4 were males. All revealed coral red fluorescence characteristic for erythrasma.

### 3.3. Microbiological Findings

Positive KOH results were noticed in 66 patients; 50 were females and 16 were males. 57 KOH mounts were highly typical of yeast species (yeast cells alone or with pseudohyphae with distinct points of constrictions) while 9 were typical of mold/dermatophytes (arthrospores and hyphae of uniform diameter). Positive bacterial growth was observed in 85 patients (64 females and 21 males). These were 47 Gram-positive cocci (36 females and 11 males) and 38 Gram-negative bacilli (28 females and 10 males) (Table 2). No combined presence of Gram-positive cocci and Gram-negative bacilli was detected in any case but both were associated with other microbial and nonmicrobial states.

### 3.4. Histopathological Findings

**Figure 1:**
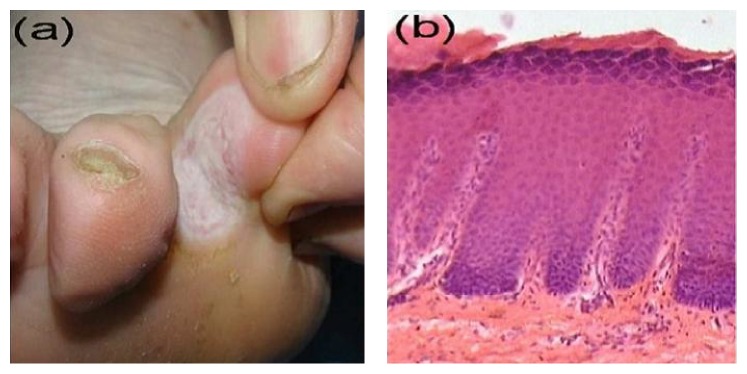
Interdigital eczema. (a) Interdigital whitish plaque with an ill-defined border. (b) Histopathological features of subacute spongiotic dermatitis (H&E, ×100).

**Figure 2:**
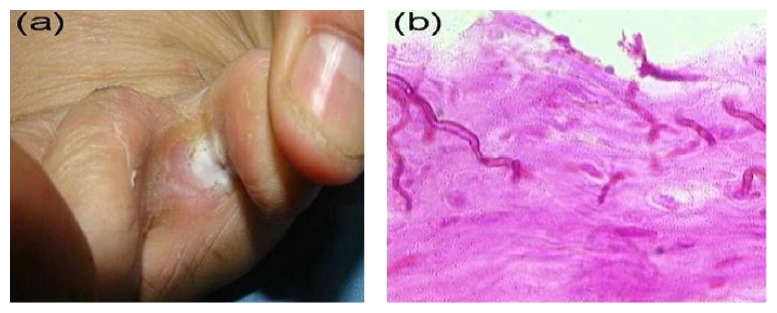
Interdigital fungal intertrigo. (a) Interdigital whitish plaques with macerated, sodden skin. (b) Biopsy from the lesion showing fungal pseudohyphae in the stratum corneum (PAS, ×400).

**Figure 3:**
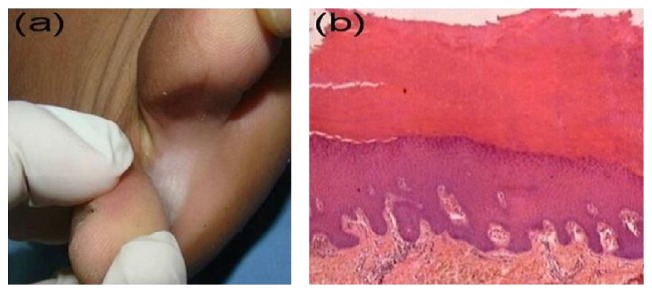
Interdigital callosity. (a) Interdigital whitish plaque with well-defined border. (b) Histopathological picture showing compact hyperkeratosis and moderate acanthosis (H&E, ×100).

**Figure 4:**
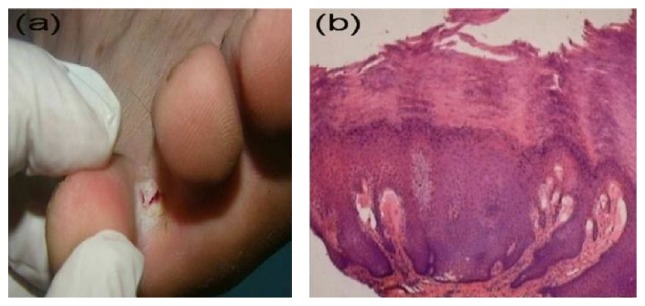
Interdigital wart. (a) Interdigital whitish plaque with well-defined border. (b) The punch biopsy shows changes characteristic for a common wart (H&E, ×100).

**Figure 5:**
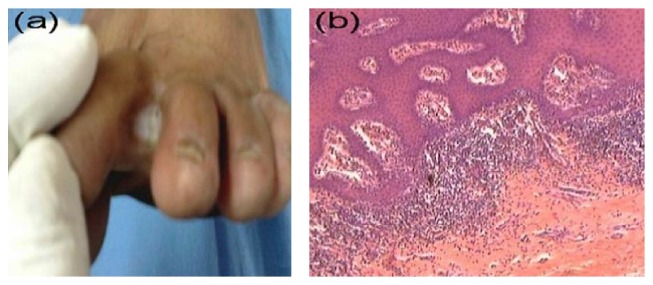
Interdigital lichen planus. (a) Interdigital whitish plaque with well-defined border. (b) The biopsy shows acanthosis, band-like lymphocytic infiltrate in the papillary dermis and vacuolar alteration of basal cell layer (H&E, ×100).

Of the 100 skin biopsy sections, 52 (40 females and 12 males) showed features characteristic for eczema (Figure 1), 25 (20 females and 5 males) showed features characteristic for fungus (Figure 2), KOH was also positive in 22 of these patients (18 yeasts and 4 dermatophytes), 19 (10 females and 9 males) showed features characteristic for callosity (Figure 3), 3 (2 males and 1 female) showed features characteristic for wart (Figure 4), and only 1 section (female) showed features characteristic for lichen planus (Figure 5) (Table 3).

Most of the patients showed more than one etiological factor for the intertrigo. 50 cases presented with 2 diseases, 38 cases presented with 3 diseases, 9 cases presented with 4 diseases, and only 3 patients showed a single disease. Positive Wood's light fluorescence (erythrasma) was associated with 14 KOH-positive cases, 15 cases with Gram-positive cocci, 6 cases with Gram-negative bacilli, 12 cases with biopsy proven eczema, 8 cases with biopsy proven fungus, and 3 cases with biopsy proven callosity. Gram-positive cocci were associated with 36 KOH-positive cases, 15 positive Wood's light fluorescence (erythrasma) cases, 10 biopsy proven fungi, 26 eczema cases, 6 callosities, and 1 wart. Gram-negative bacilli were associated with 22 KOH-positive cases, 6 positive Wood's light fluorescence (erythrasma) cases, 11 biopsy proven fungi, 20 eczema cases, 7 callosities, and 1 lichen planus.

## 4. Discussion

Foot intertrigo may present as a chronic erythematous desquamative eruption. This is often diagnosed as tinea pedis or eczematous dermatitis. However, in some patients, the macerated eruption is unresponsive to treatment with antifungals or anti-inflammatory agents [4]. Therefore, clinical and microbiological studies are suggested to assist in the selection of appropriate treatment and the prevention of important complications [3]. This study was planned to verify the etiological causes of toe web space lesions in randomly selected 100 Egyptian patients. All of the patients were adults between 18–79 years of age (mean 43. 94 yrs). This is in agreement with other similar reports [18, 19].

In our study, 72 (72%) were females; the majority of them were housewives. Household work including kitchen work, duties for cleaning, washing, caring for children and other domestic activities, and shopping may explain the increased incidence of microbial intertrigo especially tinea pedis in this sector of population. On the contrary, Ahmad et al. [19] in Pakistan reported higher rate in males (56.7%). They attributed this to wearing closed shoes most of the time in hot and humid climate. In our patients, the 4th (lateral) toe web space was the most commonly affected in both right (79%) and left (76%) foot. This is in agreement with several reports [18–20]. This could be related to anatomical considerations (potentially occluded space).

Many authors refer to web space infection as tinea pedis or “foot ringworm” and some consider it to be purely dermatophytes induced [21–23]. However, other studies have demonstrated that recovery of dermatophytes from macerated webs is low. Generally, it ranges from 7.5% to 61% [1, 24–26]. The lower incidence of dermatophyte infection in these studies may be explained by the fact that, in mixed intertrigo, bacterial production of methanethiol and other sulfur compounds can lead to inhibition of dermatophytes [1].

Depending on KOH mount, this study showed that tinea pedis was observed in 66 (66%) patients; 50 of them were females (75.76%) and 16 were males (24.24%). This agrees with Morales-Trujillo et al. [5] who declared that fungi were positive in 62.5% of 70 cases. Using KOH, Ahmad et al. [19] out of 118 cases reported higher rate of positivity where 90% had positive direct microscopy while only 50.8% had positive cultures. On the other hand, Pau et al. [27] in a study on 1568 patients reported lower rate of tinea pedis infection (14.79%). This disagreement may be due to difference in life styles (e.g., type of shoes worn), weather conditions such as humidity, and the varying number of cases in each study.

Cases with erythrasma can be diagnosed by positive Wood's lamp examination and/or Gram staining/culture [28]. In the present study, positive coral red fluorescence with Wood's light, which is characteristic for erythrasma, was found in 24% of the patients. The prevalence of erythrasma varies greatly from one study to another. Sariguzel et al. [29] reported a prevalence of 19.6% in 121 patients with interdigital foot lesions. Allen et al. [30] reported that, in 300 patients, 18.7% were determined to have erythrasma. Morales-Trujillo et al. [5] examined 73 patients, of whom 24 (32.8%) were diagnosed with erythrasma. Inci et al. [28] concluded that the rate of erythrasma was found to be 46.7% among 122 patients with interdigital foot lesions. Svejgaard et al. [31] reported a prevalence of 51.3%, (prior to military service) and 77.1% (reexamined at the end of military service) in a group of Danish military recruits.

This discrepancy may be attributed to the type of population studied, environmental conditions such as heat and humidity that increased the risk for developing erythrasma, and the methods used in diagnosis, for example, Wood's light examination, culture, and/or direct microscopy with Gram staining. In Polat and İlhan [32] study, using only Wood's lamp examination or Gram's staining resulted in 31 (42.5%) or 14 (19.2%) positive patients, respectively. Using Wood's lamp examination and Gram's staining concurrently resulted in 28 positive patients (38.4%). Interestingly, Inci et al. [28] reported no growth for* C. minutissimum* in bacteriological cultures from all patients with interdigital lesions. However, they found that using only Wood's lamp examination or Gram staining resulted in 11 (9%) and 19 positive patients (15.6%), respectively, whereas using both Wood's lamp examination and Gram staining concurrently resulted in 27 positive patients (22.1%). This suggests that bacteriological cultures have no or limited role in diagnosing erythrasma.

In this study, interdigital erythrasma was demonstrated more in females (83.3%) than males (16.7%). This finding agrees with Morales-Trujillo et al. [5] who stated that interdigital erythrasma was more common in women than men (83.3% versus 16.7%). Also, Polat and İlhan [32] stated that most of their patients with erythrasma were women (56.2%). The exact cause of this female predominance is unknown but may be attributed to occupational factors including household duties and exposure to more heat and humidity.

The interdigital space is typically colonized by polymicrobial flora. Dermatophytes may damage the stratum corneum and produce substances with antibiotic properties. Gram-negative bacteria may resist antibiotic-like substances and proliferate. This process may progress to Gram-negative foot intertrigo [4]. In this study, no combined presence of Gram-positive cocci and Gram-negative bacilli was detected in any case. Although the lack of this association is unreasonable, it could not be exactly explained. Gram-positive cocci were associated with 36 KOH and 10 biopsy proven fungi. Gram-negative bacilli were associated with 22 KOH and 11 biopsy proven fungi. Gram-negative bacterial infection may represent a secondary invasion of web space lesions. With time, in the presence of local humidity and maceration, other Gram-positive bacterial (and fungal) organisms may proliferate. So, the web space infection may represent a single or polymicrobial etiology.

Based on bacterial cultures, 47 patients (47%) were harboring Gram-positive cocci, while 38 (38%) were harboring Gram-negative bacilli. This agrees with Karaca et al. [3] who concluded that the most common pathogen was Gram-positive cocci (17.9%), followed by* P. aeruginosa* (16.7%). Also the bacteria isolated by Ahmad et al. [19] were* S. aureus* (Gram-positive cocci) in 83.4%,* P. aeruginosa* in 10%,* Proteus* spp. in 3.3%, and *β*-hemolytic streptococci in 3.3% (Gram-negative bacteria). This disagrees with Aste et al. [2] who concluded that* P. aeruginosa* was found to be the prevailing pathogen, both alone and associated with other Gram-negative bacteria (such as* E. coli*,* Proteus mirabilis*, and* Morganella morganii*) and Gram-positive bacteria.

Twenty-eight females were suffering from Gram-negative bacterial web space lesion versus only 10 males with female to male ratio 2.8 : 1. This disagrees with Aste et al. [2] who reported that Gram-negative interweb foot infection represents a male-to-female ratio of 4 : 1. Also Lin et al. [4] reported that foot bacterial intertrigo was more common in men (82%). The more prevalence in male gender can be related to the more frequent use of closed shoes for occupational and nonoccupational activities such as during the practice of sports.

Generally speaking, the routine diagnosis of interdigital lesions depends mainly upon history and clinical appearance with or without the need for direct microscopy of a KOH preparation, bacterial cultures, and/or Wood's light examination while web space biopsy is not routinely used. Yet this study declared the additional value of web space histopathology with clinical correlation in definitive diagnosis of interdigital lesions.

Despite the great clinical similarities, after clinicopathological correlations, we have diagnosed a lot of cases of interdigital eczema (52% of cases). These were clinically manifested as pruritic macerated glazed skin usually limited to the interdigital space. PAS-positive cases (interdigital fungal intertrigo) which constituted 25% of cases were clinically presented as pruritic, wet, and macerated interdigital space which usually extended to the plantar and/or dorsal surface of the foot. Some cases were associated with fungal infection of other parts of the body. Cases of callosity of toe web space (19%) were characterized by a well-defined white plaque limited to the web space. Three cases of interdigital warts were diagnosed (3%): two cases were asymptomatic while the third case was complaining of painful lesion. All of the 3 cases were associated with planter warts.

In this study, we found a healthy 49-year-old female clinically presented with pruritic interdigital lesions of 3 weeks' duration. Examination revealed bilateral and symmetrical whitish ill-defined plaques in the second and the third web spaces of both feet. Biopsy was characteristic for lichen planus. This is a very rare site for lichen planus. We think that it is important to recall such underestimated variant of lichen planus and other uncommon dermatoses in this site.

## 5. Conclusion 

Many cases of web space lesions can be overdiagnosed, underdiagnosed, or misdiagnosed. These may be caused by different conditions including eczema, fungal intertrigo, erythrasma, callosity, wart, or even lichen planus. Although fungal foot infection is common, we suggest that patients who do not respond to topical and/or systemic antifungal therapy should be reexamined for another primary or secondary dermatologic condition that may resemble pedal fungal intertrigo. The diagnostic procedures in this work can be complementary to each other and can be used as an investigative workup tailored to individual patients who have resistant to treat or have atypical interdigital lesions.

## Figures and Tables

**Table 1:** Distribution of toe web space lesions in the 100 patients.

Toe web space	Right foot		Left foot	
	Frequency	%	Frequency	%
1st space	7	7.0	8	8.0
2nd space	33	33.0	30	30.0
3rd space	62	62.0	63	63.0
4th space	79	79.0	76	76.0

**Table 2:** Microbiological findings in the 100 patients.

	Positive KOH mount	Positive bacterial cultures	
		G+ve cocci	G−ve bacilli
Female	50	36	28
Male	16	11	10
Total	66	47	38
%	66.0	47.0	38.0

**Table 3:** Frequency of histological diagnosis among the 100 patients.

Histopathological diagnosis	Frequency		Total	%
	Female	Male		
Eczema	40	12	52	52.0
Fungus	20	5	25	25.0
Callosity	10	9	19	19.0
Wart	1	2	3	3.0
Lichen planus	1	0	1	1.0
Total	72	28	100	100.0
